# Fiona Caldicott, MD, FRCPsych (Hon), DBE

**DOI:** 10.1192/bjb.2021.114

**Published:** 2022-08

**Authors:** Susan Bailey

Formerly President of the Royal College of Psychiatrists and Principal of Somerville College, Oxford, UK

**Figure:**
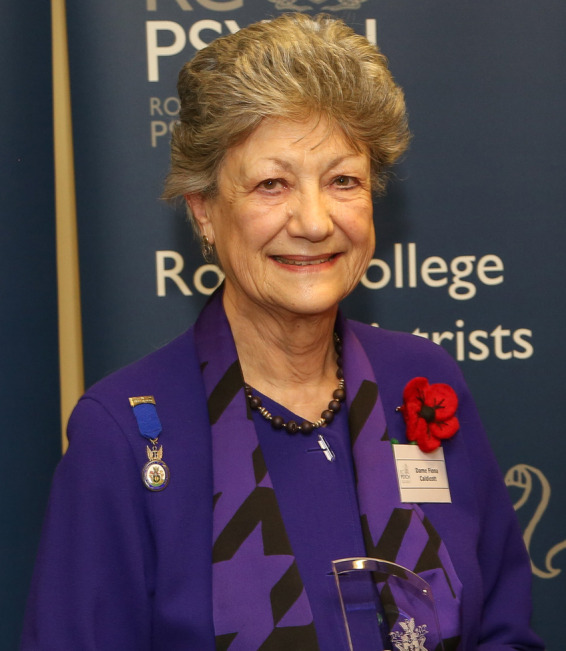

Dame Fiona Caldicott, who died on 15 February 2021 aged 80, served as the first female President of the Royal College of Psychiatrists. She went on to pursue a highly successful career as Principal of Somerville College, Oxford, as well as to chair an NHS committee that steered to a successful conclusion public policy on the highly contentious issue of the confidentiality of patient data.

Fiona's impact on the life of the College began early in the mid-1970s when she was still a senior registrar. She joined the then College Manpower Committee, becoming its Secretary and then Chair. Having trained part-time herself, she was a powerful advocate for this pathway to enable doctors with family commitments to pursue their training. Armed with accurate data, combined with superb negotiating skills and steely determination, she succeeded in persuading the government to increase the numbers of trainees in psychiatry. This was a defining moment in the life of the College and a hallmark example of how Fiona brought together her skill sets to pull a triumph out of a crisis. Then, as Sub-Dean, Fiona was a key figure in developing uniform training standards for overseas doctors working in the UK. She was elected Dean in 1990 at a time of deep personal tragedy, her son Richard dying as the result of a road accident at the age of 19. She described these two events as turning points in her life.

She was elected President of the College in 1993. At a time of major changes in postgraduate medical education, with a concurrent shift from hospital to community mental healthcare, her leadership skills enabled fellow College officers and staff to deliver on what needed to be done to improve care of and outcomes for patients. Where she shone was in working, in partnership with patient advocacy groups, with government Ministers and their civil servants in helping them to appreciate the value of the work of psychiatrists. National medical organisations were quick to appreciate her qualities and she was elected Chair of the Academy of Medical Royal Colleges.

In 1996, not long after relinquishing her role as College President, Fiona was appointed Principal of Somerville College, Oxford, 2 years after it began to admit male undergraduates. With her background in psychiatric practice with young people, she was admirably suited to taking responsibility for a community of students. During her 14-year tenure of the post of Principal, her particular contributions were in building decisions and in fundraising, particularly important for a former women's college that did not have the benefit of substantial endowments. She was greatly admired by the students, not least when she joined ‘Dame Fi's Barmy Army’ on the touchline to watch the final of the intercollegiate soccer cup.

From 1996, she chaired NHS committees on the management of patient-identifiable data. The introduction of information technology into healthcare brought challenges to the traditional principle of patient–doctor confidentiality. The reports that emerged articulated what became known as the Caldicott principles of information governance. Originally, there were six of these, but they expanded to eight after further reviews in 2013 and 2016. She recommended that every NHS trust and social services provider should appoint someone to oversee the use of confidential data. These became known as Caldicott guardians, and there are now 22 000 of them.

In later life, Fiona was much in demand to serve on public bodies. She chaired the Oxford University Hospitals NHS Foundation Trust from 2009 to 2019 and was the University of Oxford Pro-Vice-Chancellor for Personnel and Equal Opportunities from 2001 to 2010. She served on the General Medical Council, the Medical Workforce Standing Advisory Committee, the Broadcasting Standards Commission and the Nuffield Trust. She was made a Dame of the Order of the British Empire in 1996.

Fiona was born in Troon, Ayrshire, the older of the two daughters of Joseph Soesan, a barrister, and Elizabeth (née Ransley), a civil servant, who, on marriage, had to resign from her position. Fiona's childhood was spent in London, where she attended the City of London School before going on to read medicine at St Hilda's College, Oxford. While still a medical student, she married Robert, who ran a family wine business. She completed clinical studies at Birmingham Medical School. After pre-registration house jobs, she spent a period in general practice before part-time training in psychiatry and psychotherapy. In 1977, she was appointed consultant and honorary senior lecturer at the Uffculme Clinic. She was rapidly drawn into leadership roles, becoming Medical Director of the South Birmingham Mental Health Trust.

Fiona's qualities as a leader in our field were outstanding. She was regarded as leading with unswerving integrity, modesty, thoughtfulness and compassion. She did not pontificate but listened to a range of advice before reaching conclusions. She had a humanising influence not just on psychiatry, but on medical practice more generally, embedding psychological understanding into medical care. She was particularly supportive to women doctors and was instrumental in setting up the Women's Mental Health Special Interest Group at the College. She was indeed a role model for women across medicine in the way she balanced family and career. Following her, as I did, in many of the roles she had filled, I found her advice wise and inspirational. I may never have mastered her talent of how to wear a scarf with elegance and dignity, but I greatly admired the way she lived a packed life of achievement, built on the foundations of family life, love and values.

Outside her professional commitments, she was devoted to her family and enjoyed travelling with her husband on his wine-buying trips to Europe. Highly intelligent and well-informed, in 2016, she was a member of the all-women St Hilda's team that won the Christmas University Challenge.

Although from 2014 she was in poor health as a result of cancer, first of the breast and then of the pancreas, she continued in her role as National Data Guardian until shortly before her death. She is survived by her husband Robert, her daughter Lucy and her sister Judith.

